# Comparative of the effectiveness and safety of biological agents, small molecule drugs, and microbiome therapies in ulcerative colitis: Systematic review and network meta-analysis

**DOI:** 10.1097/MD.0000000000035689

**Published:** 2023-10-27

**Authors:** Jie Gao, Rui Nie, Yalan Chen, Wei Yang, Qian Ren

**Affiliations:** a Lanzhou University, Lanzhou, China; b Department of Gastroenterology, The First Hospital of Lanzhou University, Lanzhou, China; c Key Laboratory for Gastrointestinal Diseases of Gansu Province, Lanzhou University, Lanzhou, China.

**Keywords:** biological agents, FMT, probiotics, small molecule drugs, Ulcerative Colitis

## Abstract

**Background:**

Biological agents are commonly used for the first-line treatment of ulcerative colitis (UC). However, small-molecule drugs and microbiome therapies are now being used as new treatments for ulcerative colitis. We aimed to compare the relative efficacy and safety of biologics, small-molecule drugs, and microbiome therapies for the treatment of patients with moderate-to-severe ulcerative colitis.

**Methods:**

We searched the Cochrane, Embase, and PubMed databases from their inception to December 2022. RCTs that recruited patients with moderate-to-severe ulcerative colitis treated with biological agents, small-molecule drugs, and microbiome therapies. Efficacy outcomes were induction of clinical remission and mucosal healing; safety outcomes were adverse events and serious adverse events. A network meta-analysis with multivariate consistency model random-effect meta-regression was done, with rankings based on surface under the cumulative ranking curve (SUCRA) values. Higher SUCRA scores correlate with better efficacy, whereas lower SUCRA scores correlate with better safety.

**Results:**

A total of 31 RCTs comprising 7933 UC patients were included in our studies. A risk of bias assessment showed a low risk of bias for most of the included studies. Upadacitinib ranked highest for induction of clinical remission (SUCRA, 0.83) and mucosal healing (SUCRA, 0.44). Moreover, no treatments were found to increase the occurrence of adverse events compared with placebos. Ustekinumab ranked lowest for adverse events (SUCRA 0.26) and probiotic ranked lowest for serious adverse events (0·21), whereas tofacitinib ranked highest for adverse events (0·43) and upadacitinib ranked highest for serious adverse events (0·43).

**Conclusion:**

In this systematic review and network meta-analysis, we found upadacitinib to be ranked highest for the induction of clinical remission and mucosal healing, but the worst performing agent in terms of adverse events in UC patients. Probiotics were the best-performing agent for safety outcomes. More trials of direct comparisons are needed to inform clinical decision-making with greater confidence.

## 1. Introduction

Ulcerative colitis (UC) is a chronic, disabling, inflammatory disease of the colon with an increasing global prevalence and a significant effect on patient quality of life.^[1–5]^ UC often manifests between the third and fifth decade of life and is associated with an impaired health-related quality of life and a considerable economic burden.^[6,7]^

Although most people suffer a mild-to-moderate illness course, around 10% to 15% of individuals experience a severe disease course with high morbidity, recurrent flare-ups, and hospitalizations..^[8]^ Current treatments for moderately to severely active UC include corticosteroids, immunosuppressants, and targeted therapies, including tumor necrosis factor (TNF) inhibitors such as etrolizumab, vedolizumab, ustekinumab, golimumab, adalimumab, ozanimod, ozanimod, upadacitinib, and tofacitinib. Recently, studies have shown that microbiome therapies, including probiotics, synbiotics, and fecal microbiota transplant (FMT), are also a potential therapy for inflammatory bowel disease and irritable bowel syndrome.^[9,10]^

The majority of research has directly contrasted the effectiveness of placebos and biological agents with that of small molecule medications.^[11–13]^The effects of various biologics or between biologics and small molecule medications have only been directly compared in a limited number of trials.^[14–16]^Conventional meta-analysis could not obtain any conclusions about the efficacy and tolerability of treatments that were not directly compared. As a result, network meta-analysis may perform direct and indirect comparisons between different UC therapies as we are using a common comparator.

In this study, we aimed to directly and indirectly compare the efficacy of biological agents, small molecule drugs, and microbiome therapies as treatments for UC using network meta-analysis, thereby providing some guidance for clinical treatment and scientific research on the disease.

## 2. Materials and methods

### 2.1. Data sources and searches

#### 2.1.1. Search strategy.

This systematic review was conducted according to the preferred reporting items for systematic reviews and meta-analyses (PRISMA) guidelines. We established a protocol for the review, which was registered with PROSPERO prior to commencing the study (https://www.crd.york.ac.uk/prospero/, CRD42023405024).

#### 2.1.2. Study selection.

Study selection and data extraction were analyzed by 2 independent researchers, and disagreements were resolved by consulting a third investigator. We extracted data on the first author’s name, publication type, intervention type, year of trial publication, evaluation standard for treatment effect, number of participants, and number of centers involved in the trial. When available, additional data extracted about patient characteristics included age, the active duration, gender, degree of disease activity, and whether there was a recurrence of the disease. Included were full-text articles of RCTs that compared therapeutic effects on UC.

Inclusion criteria were: adults (age > 18 years) with UC (Mayo Clinic score 3 to 12, with an endoscopic subscore of 1; means of intervention were therapy using FMT, probiotics, synbiotics, small molecule drugs (ozanimod, upadacitinib, tofacitinib), or biological agents (etrolizumab, vedolizumab, ustekinumab, golimumab, infliximab, and adalimumab) with a minimum duration of 4 weeks; induction trials with a follow-up at 6 to 14 weeks and complete data on efficacy (clinical remission and mucosal healing) and safety outcomes (adverse events or serious adverse events. Exclusion criteria were articles that duplicated other publications; included animal or in vitro trials; had text in a language other than English; were case reports, reviews, meta-analyses, letters to the editor, and conference abstracts; or were publications without original data.

#### 2.1.3. Data extraction and quality assessment

Again, 2 researchers independently extracted the data, and inconsistencies resulted in a discussion until consensus was reached. The extracted data mainly included title, first author, publication year, country, study design, treatment methods, number of cases and controls included in the analysis, number of patients with clinical remission, number of patients with clinical response in induction, number of patients with AEs, and number of patients with serious adverse events (SAEs). The efficacy outcomes of this study were clinical remission and mucosal healing (intention-to-treat, ITT), and the safety outcomes were AEs and SAEs. Two researchers independently conducted risk assessments using the Cochrane Collaboration’s tool and assessed methodological quality based on the Jadad scale.

#### 2.1.4. Data synthesis and analysis

In this study, a conventional meta-analysis was carried out using RevMan 5.3. The heterogeneity of conventional meta-analysis was assessed by *I*^2^ statistic and Cochran’s Q test. *I*^2^ statistic with values > 50% or *P* < .10 for Cochran’s Q test suggested significant heterogeneity. This study used random-effects model that provided more conservative estimated effects. We calculated the relative risk (RR) and 95% confidence interval (CI) to valuate effect sizes. The symmetry of the funnel plot was used to assess the publication bias. Gemtc14.3 and Stata17.0 were used for network meta-analysis. In network diagrams, each node represents a treatment plan, and the size of nodes is related to the sample size of each treatment, which is proportional. The line between the 2 nodes indicates that there is a direct comparison between the 2 treatment regimens, and the width of lines is also proportional to the number of studies. We calculated relative risk (RR) and 95% credible intervals (95% CI) as effect statistics. We then summarized the efficacy and safety of the treatment methods used in the selected studies and calculated the absolute rates and relative ranks of different treatment methods. Heterogeneity tests, and publication bias tests were performed.

## 3. Results

### 3.1. Search results and study characteristics

**Figure 1.**
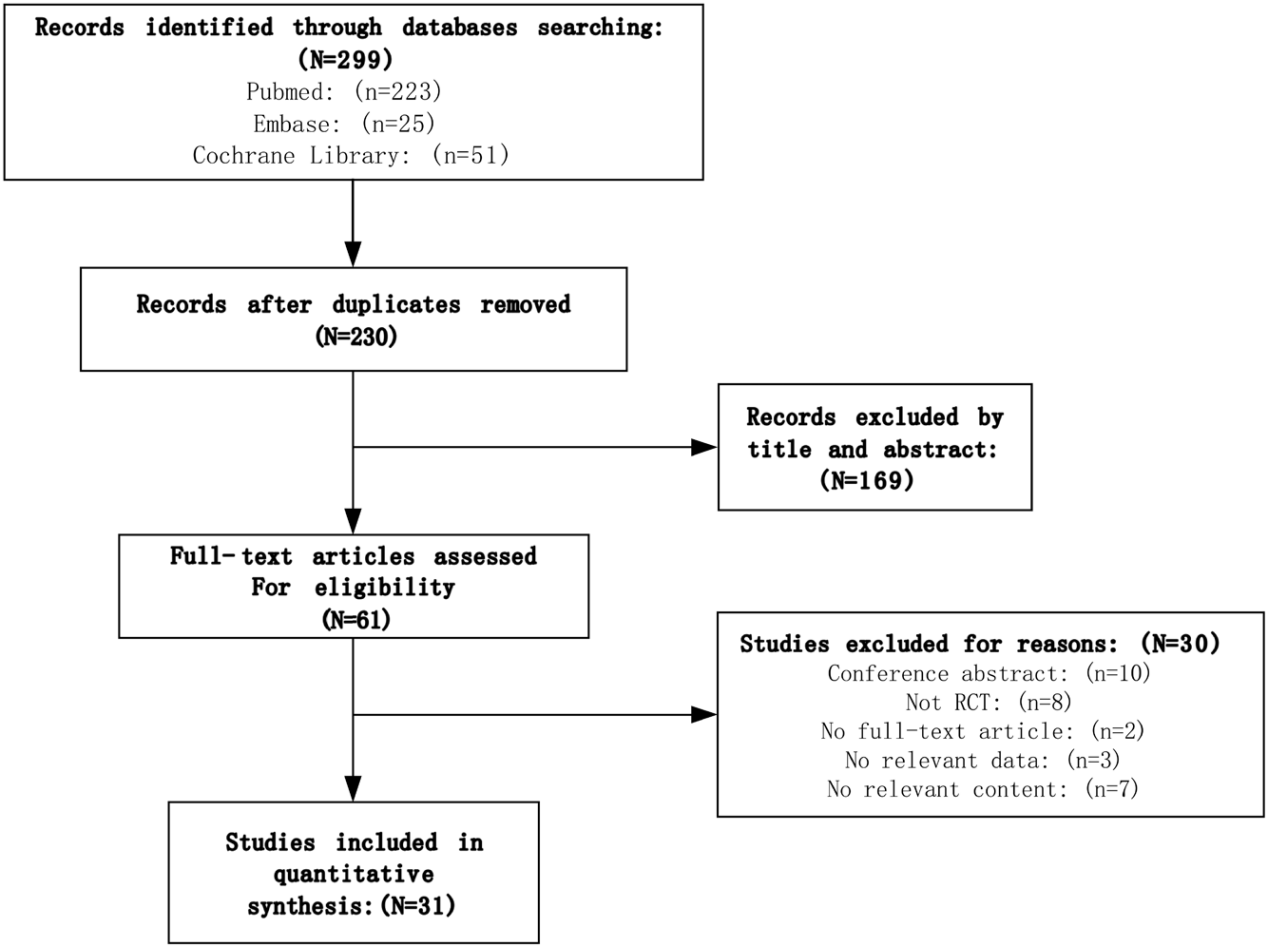
Flow diagram of evidence search and selection process.

The search strategy yielded 299 citations, of which 69 were duplicates and were removed. Of the remaining 230 records that were screened, 61 full-text articles were reviewed, of which 31 trials were eligible for inclusion. These studies included 11 different treatment types. There are 14 articles about biological agents, 6 about small molecule drugs, 7 about FMT, and 4 about probiotics. Figure 1. The characteristics of included trials are summarized in Table 1.

**Table 1:** Characteristics of the included studies.

Study	Number of people(F/M)	Intervention			Comparator	Age (yr)			Follow-up duration (wk)	ClinicalTrials. gov identifier
Biological agents										
David 2021	93/121	ETRO 105 mg 1/4 wk			Placebo/Adalimumab 160/80 mg	40.5 ± 13.5			10	NCT02163759
Severine 2022	102/112	ETRO 105 mg 1/4 wk			Placebo	43.5 ± 13.0			10	NCT02118584
Silvio 2022	147/250	ETRO 105 mg ¼ weeks			IFX5mg/kg (Weeks 0-2-6)	39.8 ± 14.3			10	NCT02136069
Rutgeerts 2015	69/83	GLM (1, 2 or 4 mg/kg 6 wk)			Placebo	41.0 ± 13.74			6	NCT00488631
William 2013	151/191	GLM 200 mg (week 0) and 100 mg (week 2)			Placebo	40 ± 13.3			8	NCT00487539
Jiang 2015	48/75	IFX 5 mg/kg (weeks 0-2-6)			Placebo	34.4 ± 14.3			8	N
Paul 2005	142/221	IFX 5 mg/kg (weeks 0-2-6)			Placebo	41.8 ± 14.2			8	NCT00036439
Taku 2015	75/140	IFX 5 mg/kg (weeks 0-2-6)			Placebo	38.9 ± 12.8			8	Japic CTI -060298
Yasuo 2013	92/181	ADA 160 mg (week 0), 80 mg (week 2), 40 mg (week 4)			Placebo	42.7 ± 11			8	NCT00853099
William 2012	200/294	ADA 160 mg (week 0), 80 mg (week 2), 40 mg (week 4)			Placebo	40.4 ± 12.8			8	NCT00408629
Walter 2010	147/243	ADA 160 mg (week 0), 80 mg (week 2), 40 mg (week 4)			Placebo	37.8 (18-75)			8	NCT00385736
Sands 2019	249/383	UST 6 mg/kg			Placebo	41.7 ± 13.6			8	NCT02407236
Brian 2013	93/112	VDZ 300 mg (weeks 0–2)			Placebo	40.3 ± 13.1			6	NCT00783718
Bruce 2019	321/451	VDZ 300 mg (weeks 0-2-6)			ADA 160 mg (week 0), 80 mg (week 2), 40 mg (week 4)	40.6 ± 13.5			14	NCT02497469
Small-molecule drugs										
William 2022	N	TOFA	10 mg	BID	Placebo		N		8	NCT00787202
William 2017	244/354	TOFA	10 mg	BID	Placebo	41.	5 ± 14.	7	8	OCTAVE 1 - NCT01465763
CP-690,550 William 2013	68/44	TOFA	10 mg	BID	Placebo	42.	8 ± 13.	7	8	NCT00787202
William 2021	257/389	OZA	1 mg	QD	Placebo	41.	6 ± 13.	5	10	NCT02435992
ACHUEVE Silvio 2022	179/293	UPA	45 mg	QD	Placebo	43	± 12.	8	8	NCT02819635
Vermeire 2021	192/324	UPA	45 mg	QD	Placebo	40	± 13.	2	8	NCT03653026
Microbiome therapies										
Haifer 2022	17/18	FMT: capsules 102.9 g			Placebo	36.4 ± 4.2			8	ACTRN12619000611123
Sarbagili 2022	14/37	Colonoscopy and retention Enema: 133.3 g			Placebo	40.4 ± 12.5			8	NCT 02734589
Březina 2021	22/23	Retention enema:500 g			Placebo	41.3 ± 8.2			12	NCT 03104036
Costello 2019	40/33	FMT: 100 g/200 mL stool			Placebo	32.6 ± 3 05			8	ACTRN12613000236796
Paramsothy 2017	34/47	FMT:37.5/150 mL stool			Placebo	37.5 ± 5			8	NCT01896635
Rossen 2015	26/22	FMT: 120 g/500 mL stool			Placebo	N			12	NCT01650038
Moayyedi 2015	31/44	FMT: 50 g/50 mL stool			Placebo	39 ± 13.		7	7	NCT01545908
Bjarnason 2019	N	Probiotic (1 mL/kg/d)			Placebo	45.4 ± 13.3			4	N
Yasushi 2015	18/28	9Bio-Three tablets/day			Placebo	43.5 ± 15.8			48	N
Katsuyoshi 2018	92/100	BFM fermented milk per day			Placebo	44 ± 12.3			48	UMIN000007593
Ajit 2009	69/88	3.6 × 1012 CFU VSL#3			Placebo	39 ± 12.8			6	N

ADA = adalimumab, BID = twice daily, EOW = every other week, ETRO = etrolizumab, GLM = golimumab, IFX = infliximab, OZA = ozanimod, QD = daily, TOFA = tofacitinib, UPA = upadacitinib, UST = ustekinumab, VDZ = vedolizumab.

### 3.2. Induction of clinical remission

**Figure 2.**
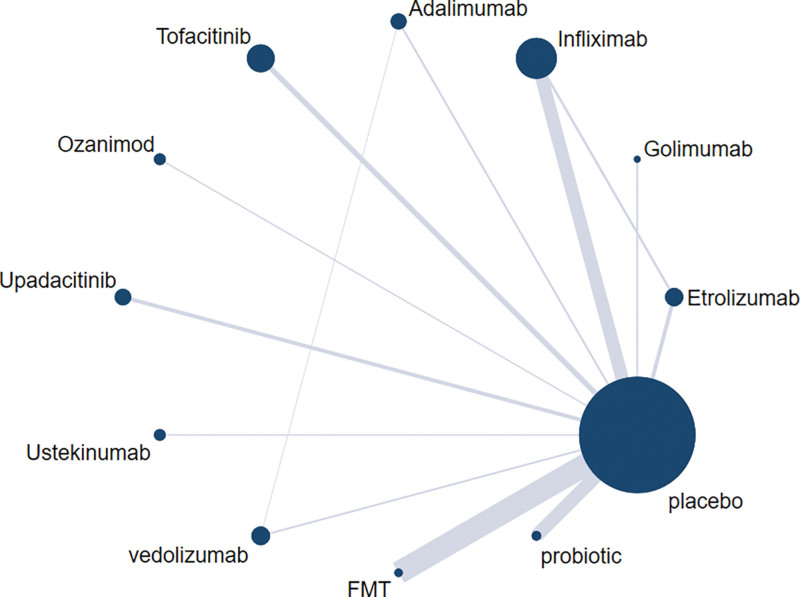
Network diagram of different treatments in clinical remission.

A total of 31 RCTs were included for efficacy analysis, and network diagrams of efficacy (clinical remission) are shown in Figure 2.

**Figure 3.**
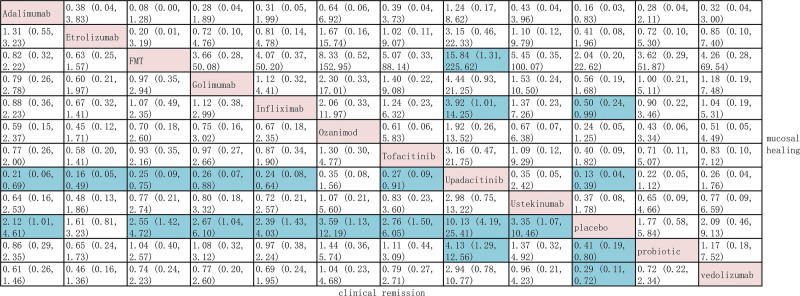
Indirect comparison for the induction of clinical remission and mucosal healing in patients with moderate-to-severe ulcerative colitis.

On direct meta-analysis, all agents were superior to placebo for induction of clinical remission, and effect size was strongest for upadacitinib (OR, 9.43; 95% CI, 5.38–16.54) and vedolizumab (OR, 3.58; 95% CI, 1.62–7.92), with substantial heterogeneity across estimates (*I*^2^ = 59%). On network meta-analysis, compared to placebo, there was moderate confidence in estimates supporting the use of infliximab, adalimumab, golimumab, vedolizumab, tofacitinib, ozanimod, upadacitinib, ustekinumab, FMT, and probiotics for the induction of clinical remission. Overall, upadacitinib (SUCRA, 0.83) was ranked highest for inducing clinical remission in patients with moderate-to-severe UC. The results from our network meta-analysis are shown in Figure 3. When evaluating the induction of clinical remission, all interventions except for ozanimod (OR 0.35, 95% CI 0.08–1.56; vedolizumab (OR 0.96, 95% CI 0.21–4.23) were significantly inferior to upadacitinib.

### 3.3. Induction of mucosal healing

When evaluating the induction of mucosal healing, all interventions except for golimumab (OR 1.74, 95% CI 0.67–4.50), adalimumab (OR 1.31, 95% CI 0.87–1.98), FMT (OR 0.52, 95% CI 0.10–2.77), and probiotics (OR 1.74, 95% CI 0.77–3.93) were significantly superior to placebo in the direct meta-analysis. Overall heterogeneity was substantial (*I*^2^ = 66%). In our network meta-analysis, a direct comparison of active treatments showed that infliximab (OR 3.92, 95% CI 1.01–14.25) and FMT (OR 15.84, 95% CI 1.31–225.62) were significantly inferior to upadacitinib for the induction of mucosal healing. Overall, upadacitinib (SUCRA, 0.44) was ranked highest. In addition, infliximab and upadacitinib were significantly superior to placebo (Fig. 3).

### 3.4. Safety of treatments

**Figure 4.**
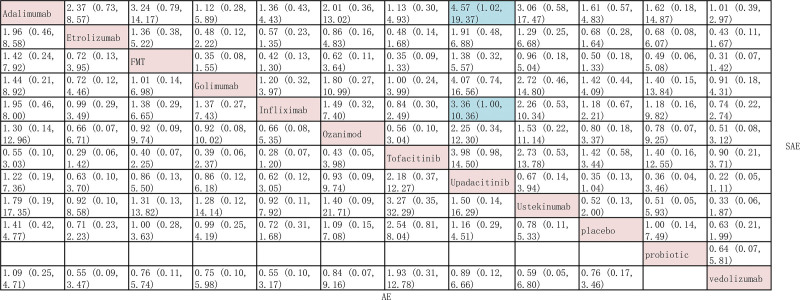
Indirect comparison for the development of adverse events and serious adverse events in patients with moderate-to-severe ulcerative colitis.

In our network meta-analysis, no difference in adverse events and serious adverse events was observed between active interventions. (Fig. 4) Upadacitinib ranked highest when considering adverse events (SUCRA 0.43) and serious adverse events (SUCRA 0.43). Ustekinumab ranked lowest for adverse events (0.26) and probiotics ranked lowest for serious adverse events (0.21).

## 4. Discussion

We identified numerous significant findings in our systematic review and network meta-analysis that included direct and indirect evidence from 31 studies. First, all biological agents, small molecule drugs, and microbiome therapies were significantly better than placebo in terms of inducing clinical remission, and through our network meta-analysis, we found that upadacitinib ranked highest for the induction of clinical remission and mucosal healing. In terms of safety, probiotics were ranked as the safest drug in terms of adverse events. In addition, a novel finding of this study was that ustekinumab ranked lowest for serious adverse events. We cannot determine if probiotic safety was superior to ustekinumab safety in terms of adverse events since the number of probiotic RCTs was modest and the number of adverse events was not recorded.

Mucosal healing has emerged as an important therapeutic endpoint in the management of inflammatory bowel diseases.^[17–21]^However, few clinical trials have included it as an outcome thus far. In this systematic review and network meta-analysis, we confirmed that anti-TNF biologics and small molecule drugs are effective in inducing mucosal healing in UC. Danese examined mucosal healing as an endpoint for UC,^[22]^ and demonstrated the superiority of infliximab over adalimumab for the induction of clinical response and mucosal healing. However, our research confirms that upadacitinib is significantly superior to infliximab for the induction of mucosal healing. In addition, the definition of histological remission was proposed by IOIBD as early as 2014. Recently, the European Crohn′s and Colitis Organization (ECCO) statement also recommended the most stringent concept of histological remission and the definition of histological remission after UC treatment. Christensen et al compared patients with different histological activities and showed that those with histological healing had a lower risk of recurrence than those with histological remission.^[23]^In this regard, ECCO guidelines recommend histological remission as a treatment for UC goals.^[24]^However, the number of studies that included histological mitigation as an outcome was too small for secondary analysis in this paper.

The discovery of oral small molecule drugs has become an area of interest in the search for more effective and well-tolerated long-term treatments for UC.^[25]^This is the second small molecule drug after tofacitinib to be approved for the treatment of UC.^[15]^These findings support previous indirect treatment comparison network meta-analyses and observational comparative effectiveness studies that found upadacitinib to have better efficacy and effectiveness than adalimumab, etrolizumab, tofacitinib, and golimumab. Upadacitinib12 is an oral, selective, small-molecule JAK inhibitor designed to inhibit JAK1 more than JAK2, JAK3, and tyrosine kinase 2.^[15]^ Upadacitinib is an oral, selective, small molecule JAK inhibitor engineered to have greater inhibitory effects on JAK1 than on JAK2, JAK3, and tyrosine kinase 2.^[26]^ As an oral small molecule drug, upadacitinib may offer various additional benefits to biologic therapy, including increased treatment adherence and a lack of immunogenicity. Unlike tofacitinib, upadacitinib is a preferential Janus kinase-1 inhibitor, and upadacitinib ranked first in many analyses, but it is important to note that complete results from these trials are not yet available.

Ozanimod’s effectiveness as a small-molecule medicine for the treatment of UC was further supported by our investigation. Ozanimod is a sphingosine-1-phosphate (S1P) receptor modulator that binds with high affinity to S1P subtypes 1 and 5 (S1P1 and S1P5), leading to the internalization of S1P1 receptors in lymphocytes and the prevention of lymphocyte mobilization to inflammatory sites.^[27–29]^ It is now approved in the United States for the treatment of adult patients with moderately to severely active UC and patients who have had an inadequate response or loss of response to conventional therapy or one of the EU biologics. In randomized controlled clinical trials, ozanimod reduced the levels of the intestinal inflammatory markers fecal calprotectin (FCP) and fecal lactoferrin (FLF) during induction therapy in patients with UC; these reductions were maintained through maintenance therapy.^[30]^

UC patients suffer from recurrent disease and their quality of life is significantly reduced.^[31]^ For patients with severe disease activity, clinical trials have shown that corticosteroids are effective.^[32]^ It has been reported that immunosuppressive agents can alleviate the disease and reduce the use of corticosteroids.^[33]^However, the medical options for corticosteroids and immunological formulations are expensive and have significant toxicity. Corticosteroids can cause acne and weight gain and can even lead to opportunistic infections that can exacerbate the condition.^[34]^Long-term use of corticosteroids may increase the risk of osteoporosis, and long-term use of immunosuppressants has been associated with the development of cancer.^[35]^ Probiotics, prebiotics, synbiotics, or FMT are becoming increasingly important for inducing active UC remission.^[36]^

FMT is a new treatment option that differs from other pharmacological treatments. Based on network meta-analysis, the efficacy of FMT is comparable to that of biologics and small-molecule drugs, which may provide a new avenue for UC treatment. In a recent long-term safety and efficacy study with a follow-up period of 1 to 5 years, the results suggest that FMT should be a safe and promising treatment for UC.^[37]^Most people still consider FMT to be an experimental therapy. Further studies are needed to establish the optimal approach, efficacy and safety of FMT to make such a comparison more convincing. Our analysis showed no significant differences in therapeutic efficacy between FMT and probiotic preparations in terms of clinical remission and mucosal healing. Infliximab was found to be the most effective drug in terms of inducing clinical remission and endoscopic improvement in an initial network meta-analysis by Singh et al, with vedolizumab ranking second.^[38]^ This work, which will be updated beginning in 2020, again demonstrates that infliximab ranked first in clinical remission and endoscopic improvement in patients with biologic hubs, with ustekinumab and tofacitinib having the highest efficacy in patients previously exposed to biologics.^[16]^A recent net meta-analysis indicated that upadacitinib was the best-performing agent for the induction of clinical remission (the primary outcome) and was the worst performer in terms of adverse events in patients with moderate to severe UC.^[15]^Meanwhile, several meta-analyses on probiotics and FMT over the past few years have pointed out that significantly higher clinical and endoscopic remission rates are observed with FMT than with control treatments. However, the current study was unable to determine the effect of probiotics in terms of clinical remission in patients with active UC. This is because the studies had very few participants and were not conducted using reliable methods.^[9,10,39]^

In addition to the inherent limitations of reticulated meta-trials, our analysis also has limitations. Thorough comparative analyses of all drugs were limited to trials of induction therapy, making it difficult to assess their effectiveness due to differences in the design of maintenance therapy trials. Second, we only statistically analyzed the incidence of clinical remission, mucosal healing, and AE in this study and did not examine clinical improvement or histological remission. Therefore, the overall treatment effect of UC needs to be further determined. Furthermore, although probiotics and ustekinumab showed a good safety profile in our study, the lack of completeness of adverse event data requires further evaluation of safety. In addition, we did not perform a statistical analysis due to the lack of data from studies evaluating the cost-effectiveness of small molecule drugs, biologics, and microorganisms for the treatment of UC. The included studies also differed in the clinical diagnostic criteria and clinical subtype classification of UC, which may lead to inaccurate conclusions. Finally, we included only one article on ozanimod and ustekinumab, which may have led to biased results due to the small sample size. To confirm the efficacy and safety of small molecule drugs, a multicenter randomized controlled clinical trial with large sample size is needed.

Despite its limitations, this network meta-analysis provides new evidence. To our knowledge, there are no comparative studies on microbial treatments, biologics and small molecule drugs. Direct evidence suggests that, in terms of clinical remission, all included treatments were due to placebo, indirect evidence suggests that biologics remain highly effective for ouabainide and that small molecule drugs have the best therapeutic effect but have a poorer safety profile. In addition, ozanimod and vedolizumab may also be effective drugs for the treatment of UC. The efficacy of FMT and probiotics may provide some options for the treatment of UC. In this study, the best safety profile was for probiotic therapy, and upadacitinib was the most effective drug for patients with moderate to severe UC in terms of clinical remission and mucosal healing during the induction period, with ozanimod ranking second. However, the safety of upadacitinib remains to be investigated. Probiotic agents were all more effective than placebo but not as effective as small molecule drugs and biologics, but they had the best safety profile.

## Author contributions

**Conceptualization:** Jie Gao.

**Data curation:** Jie Gao, Yalan chen.

**Formal analysis:** Jie Gao.

**Funding acquisition:** Qian Ren.

**Project administration:** Wei Yang.

**Resources:** Rui Nie.
